# Optimized Callus Subculture and Multi-Omics Analysis Reveal Somatic Embryogenesis Bottlenecks in the Cold-Resistant Rubber Tree Clone GT1

**DOI:** 10.3390/plants15192924

**Published:** 2026-09-24

**Authors:** Chenrui Gu, Xinran Ou, Xianfeng Yang, Xiaochuan Gu, Quannan Zhou, Jing Cheng, Shichao Xin, Ji Li, Tiandai Huang

**Affiliations:** 1 State Key Laboratory of Tropical Crop Breeding, Rubber Research Institute, Chinese Academy of Tropical Agricultural Sciences, Key Laboratory of Biology and Genetic Resources of Rubber Tree, Ministry of Agriculture and Rural Affairs, Key Laboratory for Cultivation & Physiology of Tropical Crops of Hainan Province, State Key Laboratory Incubation Base for Cultivation & Physiology of Tropical Crops, Haikou Innovative Key Laboratory for Tropical Crop Seedlings, Haikou 571101, China; guchenrui@catas.cn (C.G.); 24220951310117@hainanu.edu.cn (X.O.); shouyiweida@163.com (X.Y.); guxiang28@163.com (X.G.); zhouquannan2004@163.com (Q.Z.); jordanafs@163.com (J.C.); scxin@catas.cn (S.X.); 2 Sanya Research Institute of Chinese Academy of Tropical Agricultural Sciences, Sanya 572024, China; 3 School of Tropical Agriculture and Forestry, Hainan University, Haikou 570228, China

**Keywords:** *Hevea brasiliensis*, somatic embryogenesis, epigenomics analysis, callus subculture, gene regulatory network

## Abstract

The rubber tree (*Hevea brasiliensis* (Willd. ex A. Juss.) Müll. Arg.) is the predominant global source of natural rubber; however, its cultivation is largely confined to tropical regions due to chilling sensitivity. Although the chilling-tolerant clone GT1 holds considerable promise for expanding cultivation latitudes and serving as a genetic transformation recipient, its utility is severely compromised by a recalcitrant somatic embryogenesis (SE) capacity relative to the elite clone Reyan 7-33-97 (R73397). Despite prior media optimization efforts, an efficient callus subculture system for GT1 remains elusive, and the molecular basis of its SE recalcitrance is poorly characterized. Here, we establish a repeated GT1 callus subculture system using a pulsed plant growth regulators regimen and integrated comparative transcriptomics, whole-genome bisulfite sequencing (WGBS), and Assay for Transposase-Accessible Chromatin sequencing (ATAC-seq) with Tn5 footprinting to elucidate the regulatory bottlenecks distinguishing GT1 from R73397. Our results suggest that, although the GT1 callus has not completely lost the expression of genes that characterize the tendency to develop into embryos, it does not complete the morphogenetic transition into globular embryos, possibly due to the reduced levels of transcription factors critical for embryo patterning. The epigenetic analyses indicate that the differential expression of these regulators is not tightly coupled with DNA methylation or chromatin accessibility, but reveal distinct transposable element silencing mechanisms between the two genotypes. Furthermore, construction of a hierarchical TF-DNA binding network predicts the positions of several transcription factors, which have previously been reported to positively regulate somatic embryogenesis within the gene regulatory network. Collectively, this work overcomes the technical bottleneck associated with multiple subcultures of GT1 callus tissue and provides a multi-omics framework for identifying the precise genetic targets to facilitate SE in chilling-tolerant rubber tree breeding.

## 1. Introduction

The rubber tree (*Hevea brasiliensis* (Willd. ex A. Juss.) Müll. Arg.) is the primary source of natural rubber, but its cultivation is geographically restricted to tropical regions. The popularization of cold-tolerant varieties could partially expand the geographical range and scale of rubber plantations. The primary clone GT1 and its derived secondary clones exhibit robust cold tolerance [1,2]. Employing these cold-tolerant clones as recipient materials for genetic engineering would provide a superior baseline for cold resistance. However, this approach is severely hindered by the exceptionally low somatic embryogenesis rate of GT1 [3]. In contrast, the anther callus induction and genetic transformation systems of clone Reyan 7-33-97 (R73397) are well established, rendering it the predominant recipient clone for rubber tree genetic engineering [4].

Although callus induction of GT1 can currently be achieved through various methods [3,5], efforts to establish efficient callus subcultures and enhance their somatic embryogenesis rate by optimizing the medium components and plant growth regulators (PGRs) ratios have yielded unsatisfactory results. To overcome the recalcitrance of GT1 to somatic embryogenesis, it is necessary to comprehensively compare its transcriptional profiles and gene regulatory networks during somatic embryogenesis with those of the embryogenic clone R73397, thereby unraveling the key determinants underlying the low embryogenesis rate of GT1.

Following seed germination, plant somatic cells generally cease to spontaneously initiate embryogenesis. Upon auxin induction, transcriptional regulators from families such as *WRKY*, *CAMTA*, and *TCP* drive the dedifferentiation of somatic cells into callus cells [6,7], which subsequently redifferentiate into somatic embryos or adventitious shoots through a complex reprogramming of gene expression. Previous studies have indicated that the differentiation of somatic embryos or adventitious shoots is orchestrated by hallmark transcription factors, including *BABY BOOM* (*BBM*), *WUSCHEL-related homeobox* (*WOX*), *LEAFY COTYLEDON* (*LEC*), and *AGAMOUS-LIKE15* (*AGL15*) [8,9].

Artificially elevating the transcript levels of morphogenetic regulators *BBM* and *WUS2* enhances the genetic transformation and gene editing efficiency in monocots, such as maize (*Zea mays* L.) [8], sorghum (*Sorghum bicolor* L.) [10], and wheat (*Triticum aestivum*) [11], by promoting the proliferation of scutellum-like structures on callus cells. *BBM* encodes an AP2/ERF transcription factor expressed in both somatic and zygotic embryos, and its ectopic expression promotes somatic embryo formation in *Arabidopsis thaliana* (L.) Heynh [12]. In dicots, the functional orthologs of maize *ZmWUS2* are *WOX2* and *WOX4*. In *Arabidopsis thaliana*, the expression of *AtWOX2* and *AtWOX3* is directly activated by *LEC2* to trigger somatic embryogenesis [6]. These *WOX* genes likely determine meristem developmental patterns by regulating the expression of auxin biosynthesis genes, such as *YUC4* and *TTA1* [13]. Additionally, *WOX5* and *WOX7* form functional complexes with *SHOOT MERISTEMLESS* (*STM*), maintaining meristematic cell activity by modulating *PLETHORA1–2* (*PLT1–2*) and Auxin Response Factors (ARFs) [14,15]. *LEC1* and *LEC2*, which belong to the nuclear factor Y (NF-Y) family and are embryo-specifically expressed, function in concert with VP1-/ABI3-like B3 transcription factors encoded by *FUSCA* (*FUS*) [12]. Overexpression of *LEC* genes significantly enhances somatic embryogenesis efficiency. Exhibiting an expression pattern similar to *LEC1*, the *Somatic Embryogenesis Receptor-like Kinase* (*SERK*) gene encodes a leucine-rich repeat receptor-like kinase (LRR-RLK). Its expression level is strictly positively correlated with embryogenic events, serving as a marker for embryogenic cells [16]. Overexpression of *SERK* in *Arabidopsis thaliana* increases the somatic embryogenesis capacity three- to four-fold [17]. Similarly, ectopic expression of *AGAMOUS-LIKE15* (*AGL15*) or *AGL18* [18,19] also promotes somatic embryogenesis in *Arabidopsis thaliana* and soybean (*Glycine max* (L.) Merr.) [20,21]. Furthermore, several genes outside the core regulatory hierarchy of somatic embryogenesis play crucial roles in redifferentiation. For instance, *MYB118* and its homolog *MYB115* positively regulate *LEC1* expression [22]. *WIND1* (also known as *DERB1*), an AP2/ERF transcription factor, mediates tissue responses to mechanical wounding and cytokinin, thereby regulating both dedifferentiation and redifferentiation [23]. Additionally, *ESR* genes interact with totipotency factors, such as *LEC1*, *LEC2*, and *BBM,* to modulate their activities [23].

In the rubber tree, elevating the transcript levels of *GROWTH-REGULATING FACTOR 4* (*GRF4*) and its interacting partner *GRF-INTERACTING FACTOR 1* (*GIF1*) can further enhance the somatic embryogenesis rate of clone R73397 [24]. Three newly identified *MADS-box* genes exhibit specific expression during somatic embryogenesis, although their crucial roles in this process remain to be elucidated [25]. Comparative transcriptomic analyses have revealed differential enrichment of genes within the MAPK signaling pathway—associated with ethylene signal transduction—between the calluses of clones YY73477 and RK628. The resulting variations in the expression profiles of somatic embryogenesis-related genes may account for the divergence in their embryogenesis rates [26].

In this study, we optimize the subculture strategy for GT1 callus, achieving the generation of high-quality callus through the alternating application of PGR combinations. Through comparative transcriptomic analysis of GT1 and R73397 calluses, integrated with ATAC-Tn5 footprinting, we predict the transcription factor regulatory networks underlying somatic embryo initiation in rubber tree calluses and identify the key transcription factors with promising application potential.

## 2. Results

### 2.1. Subculture Strategy for GT1 Anther-Induced Callus

Given the advanced age of the field-grown experimental materials, we first verified their clonal identity through whole-genome resequencing (WGS). We profiled the SNPs/InDels across several rubber tree clones (CATAS8-333, GT1, PR107, RRIM600, RY879, ReKen628, and YY774) and performed a principal component analysis (PCA). Our selected sample exhibited the highest genetic similarity to the published GT1 reference data, surpassing even its progeny clone YY774 (Figure 1). Conversely, its similarity to other common cultivars grown in Hainan was notably low. Based on these genomic data, we conclusively confirmed that the experimental material used in this study is clone GT1. The detailed PC1–PC9 data are provided in Supplementary Table S1.

We initially induced GT1 anthers using the anther callus induction method and medium previously established for R73397 [27]. GT1 is a male-sterile clone; its male flowers typically wither and abscise before reaching 2 mm in length, meaning that only smaller anther explants can be harvested compared to R73397. These male flowers lack the typical yellow appearance, and their anthers are darker in color and visibly smaller than those of R73397 (Figure 2A–D). Following a 30-day dedifferentiation induction and a 15-day somatic embryo induction, anthers from R73397 successfully developed into a vigorously growing callus, while the callus of GT1 grew slower and possessed a relatively firm texture (Figure 2E,F). However, after somatic embryo induction, the GT1 callus gradually underwent browning, becoming severely browned and entirely necrotic after 80–85 days (Figure 2G,H).

We subsequently induced a GT1 anther callus using GT1 callus induction medium (MS medium supplemented with vitamins 4.4 g/L, Phytagel 2.3 g/L, Sucrose 70 g/L, 6-Benzylaminopurine 1 mg/L, 2,4-Dichlorophenoxyacetic acid 0.5 mg/L, and Gibberellin A3 0.5 mg/L; pH = 5.8) which was further improved based on the formulation previously published by Li et al. [5]. After 30 days, a vigorously growing GT1 anther callus was obtained. Subculturing this callus with GT1 subculture medium (MS medium supplemented with vitamins 4.4 g/L, Phytagel 2.3 g/L, Sucrose 70 g/L, Kinetin 2 mg/L, α-Naphthaleneacetic acid 2 mg/L, and Thidiazuron 0.2 mg/L; pH = 5.8), which was further improved based on the formulation previously published by Li et al. [5], yielded a proliferating but yellowed callus after 30 days. This outcome was entirely consistent with the findings of Li et al., indicating that this approach fails to support the long-term subculture and proliferation of GT1 anther callus. To overcome this, we alternated the culture of the yellowed callus, switching back to the GT1 callus induction PGR formulation, which yielded highly satisfactory results. The yellowed callus resumed rapid proliferation, turned light yellow, and developed a relatively friable texture (Figure 2I). By repeatedly alternating between these two PGR formulations, we successfully achieved the multiple times proliferation of GT1 anther callus with a satisfactory proliferation proportion (Figure 2J). The biomass accumulation rate and proliferation proportion of GT1 callus under the new tissue culture strategy was roughly estimated by measuring the fresh weight of the callus (Supplementary File S1).

### 2.2. Comparative Transcriptomic Analysis of GT1 and R73397 Calluses at Two Distinct Developmental Stages

We subsequently performed transcriptomic sequencing on four types of calluses. Due to the slow development of GT1 callus tissue in the medium previously established for R73397, we had to perform RNA-Seq using GT1 anther callus tissue induced in the new medium, despite the differences in the proportions of PGRs between the two media. GT1-induce is the GT1 callus cultured on GT1 callus induction medium (Figure 2I); R73397-induce is the R73397 anther callus induced using the previously published dedifferentiation induction medium [27] (having an appearance almost identical to that shown in Figure 2E); GT1-rediff is the GT1 anther callus subjected to somatic embryo induction for 15 days using the published embryogenic induction medium (Figure 2F) [27]; and R73397-rediff is the R73397 anther callus subjected to somatic embryo induction for 15 days using the published embryogenic induction medium (Figure 2E) [27]. Three biological replicates were performed for each sample, yielding a total of 211 Gb of clean data, with Q30 ≥ 90% and Q20 ≥ 95%. The average mapping rate to the reference genome (GCF_030052815.1_ASM3005281v1) was 95.78% (with all samples exceeding 94%). The principal component analysis (PCA) (Figure 3A) revealed distinct segregation in the gene expression profiles among the four types of calluses, while maintaining high reproducibility within each group.

Applying thresholds of *Padj* ≤ 0.01 and |log2FC| ≥ 1.0, we identified 8561 DEGs between GT1-induce and R73397-induce, and 14,723 DEGs between GT1-rediff and R73397-rediff (Figure 3B). The results of KEGG enrichment analysis for both sets of DEGs are provided in Supplementary Files S2 and S3 and Supplementary Tables S2 and S3.

We performed a Gene Ontology (GO) enrichment analysis on the upregulated and downregulated genes within each DEG set separately. If the differentially expressed genes (DEGs) between the two callus types during the callus induction stage (induce) were primarily driven by the different PGR formulations, they should be enriched in typical hormone-responsive biological processes, such as ‘auxin response’. However, the enriched biological processes for the DEGs between GT1-induce and R73397-induce were predominantly concentrated in development-related pathways (Figure 4(A—left; B—left)). The “post-embryonic development” process was significantly enriched among the upregulated genes of R73397 during the callus induction stage, rather than the “embryo development” process (Figure 4A). Although these observations could be confounded by different PGR ratios, they nonetheless indicate that the extremely low somatic embryogenesis rate in GT1 may be attributed to an unsuccessful transition from a single cell to an embryonic structure. The “flower development” process also showed enrichment, likely resulting from the residual expression of anther tissue-specific genes. We hypothesize that because GT1 exhibits incomplete anther development, it lacks the expression of this subset of genes (Figure 4A).

Despite being cultured on identical media and PGR formulations, a higher number of DEGs were observed between GT1-rediff and R73397-rediff. This further shows that the different GT1 and R73397 genotypes have a big impact on gene transcription. During both the callus induction and somatic embryo induction stages, the highly expressed genes in R73397 were significantly enriched in the “anatomical structure development” and “multicellular organism development” pathways (Figure 4A). These biological processes are critical for the development of complex embryonic structures.

To identify potential somatic embryogenesis inhibitors, we focused on the genes highly expressed in GT1 by performing an enrichment analysis on the two aforementioned sets of downregulated genes (i.e., genes showing higher expression in GT1) (Figure 4B). During the callus induction stage, the “programmed cell death” process was more active in GT1. Although the programmed cell death of specific cells facilitates the shaping of the embryonic body and the segregation of embryogenic from non-embryogenic cell clusters, we postulate that this phenomenon is more likely a consequence of clearing senescent cells induced by the “response to stress” process in GT1. This explains why the continuous use of a single medium fails to sustain its cell proliferation. During the somatic embryo induction stage, the “reproductive process” was more active in GT1. Generally, this biological process indicates that plant tissues have a strong propensity for somatic embryogenesis, which is in contrast to the very low somatic embryogenesis rate of GT1. We then conducted a cross-stage differential gene analysis between GT1-induce and R73397-rediff, followed by a GO enrichment analysis of the downregulated genes (i.e., those highly expressed in GT1) (Figure 4C). Contrary to our expectations, the “embryo development” pathway was significantly enriched in this set of downregulated genes (Figure 4C).

To identify the transcription factors that activate both the “post-embryonic development” and “anatomical structure development” processes (i.e., those positively regulating the entire transition of embryogenic cells into globular embryos), we annotated the intersecting genes (1583 genes) between the two sets of upregulated genes. Based on predictions from the PlantTFDB [28], 127 of these intersecting genes were annotated as transcription factors (key-TFs). The expression levels of these key-TFs are provided in Supplementary Figures S1 and S2.

### 2.3. Differential Epigenetic Modifications Between GT1 and R73397

Although occasional somatic embryogenesis occurs during tissue culture of GT1 [5], the induction rate is extremely low [3]. This indicates that the GT1 genome retains a complete repertoire of genes required for somatic embryogenesis; however, because the callus is derived from incompletely developed anthers, these essential genes fail to be properly expressed. We hypothesized that anthers with incomplete development failed to undergo proper reprogramming of genomic epigenetic imprints, resulting in the presence of epigenetic modifications within the callus that impede somatic embryo development. To test this, we performed whole-genome bisulfite sequencing (WGBS) on GT1-rediff and R73397-rediff. Three biological replicates were performed for each callus type, yielding a total of 470 Gb of clean data with Q30 ≥ 90% and Q20 ≥ 95%. After retaining the perfectly aligned paired-end reads (one forward and one reverse mate), the average mapping rate to the reference genome was 98.31%. A principal component analysis (PCA) of the genomic methylation profiles revealed distinct segregation and high intra-group reproducibility for all three methylation contexts (CG, CHG, and CHH) between the two types of calluses (Figure 5A). Within the gene regions, the average methylation levels of the two callus types were nearly identical, exhibiting a typical distribution pattern characterized by reduced methylation at the transcription start site (TSS) and transcription end site (TES) (Figure 5B). However, CHH methylation within the transposable element (TE) regions of GT1 anther callus was significantly hypo than in R73397 (Figure 5C), suggesting a potentially weakened methylation pathway for TE silencing. The analysis using Metilene identified 849, 902, and 484 differentially methylated regions (DMRs) in the CG, CHG, and CHH contexts, respectively. However, the differential expression of key transcription factors showed no strong correlation with these DMRs (Figure 5D). Among the 127 key transcription factors identified earlier, only five harbored differential methylation modifications (Figure 5D,E).

We subsequently performed ATAC-seq to assess the chromatin accessibility of the two types of calluses. Two biological replicates were performed for each callus type, yielding a total of 131 Gb of clean data with Q30 ≥ 90% and Q20 ≥ 95%. After retaining the perfectly aligned paired-end reads, the average mapping rate to the reference genome was 96.96%. A principal component analysis (PCA) of the accessible chromatin profiles revealed distinct segregation and high intra-group reproducibility between the two types of calluses (Figure 6A). Within the gene regions, the average chromatin accessibility levels of the two callus types were nearly identical, exhibiting a typical distribution pattern characterized by a peak at the transcription start site (TSS) (Figure 6B). In contrast, within the transposable element (TE) regions, the average chromatin accessibility levels differed significantly between the two callus types, with R73397 exhibiting noticeably higher TE accessibility than GT1 (Figure 6C). The differential expression of the key transcription factors showed only a weak correlation with chromatin accessibility. The analysis using PePr identified 1368 more accessible and 12,177 less accessible differentially accessible regions (DARs) in R73397 compared to GT1. Among the 127 key transcription factors, only 22 harbored DARs, the majority of which exhibited reduced accessibility in R73397 (Figure 6D,E).

### 2.4. TF-DNA Binding Network of Key Transcription Factors Underlying Biological Processes

Another major application of ATAC-seq is the prediction of transcription factor binding motifs based on Tn5 transposase footprints. Given that the key-TFs were expressed at low levels or were almost undetectable in the GT1 callus, we utilized the ATAC-seq data of R73397 to predict which key-TFs bind to the DEGs involved in the “anatomical structure development” process. Based on the predicted TF-DNA binding network, we stratified these transcription factors into three hierarchical layers (Figure 7A) and highlighted those previously reported to explicitly promote secondary somatic embryogenesis (Figure 7A,B). All 127 key-TFs exhibited direct or indirect binding associations with this biological process, and 55 of them were themselves annotated within the “anatomical structure development” process. This further substantiates that the identified 127 key-TFs constitute the core gene set responsible for the differential somatic embryogenesis rates between GT1 and R73397. *LOC110633278* (WOX8) and *LOC110635766* (NFY-A-3) are known positive regulators of somatic embryogenesis that harbor DARs. We validated their high expression levels in R73397 using quantitative reverse transcription polymerase chain reaction (qRT-PCR) (Figure 7C,D). However, their differential chromatin accessibility sites all corresponded to less accessible DARs in R73397, positioned immediately adjacent to the TATA box (Figure 7C,D).

## 3. Discussion

Cold stress confines the worldwide distribution of plant growth [29], especially for tropical plants. The application of the cold-resistant rubber tree clone GT1 in genetic engineering breeding is severely limited by its extremely low rate of somatic embryogenesis. Even the genetic transformation aimed at improving somatic embryogenesis rates inherently requires a sufficient quantity of vigorously growing callus as the recipient material. Although the foundational research by Li, M. X. et al. [5] did not completely resolve the issue of callus subculture and proliferation in GT1, it provided a valuable starting point for our study. Building upon this previous work, we utilized a modified MS medium containing vitamins and achieved the repeated subculture and proliferation of GT1 callus by alternating between two different PGRs. Consistent with our enrichment analysis, the “response to stress” and “programmed cell death” processes were more active in the GT1 callus, leading to increased cellular browning and death. This explains why the continuous use of a single medium fails to support long-term subculture. We hypothesize that the alternating PGR treatments disrupt these detrimental processes. The intermittent application of the TDZ signal continuously prevents cell death and reactivates cell division and proliferation, while simultaneously preventing the browning and necrosis that result from prolonged TDZ exposure [30].

It should be noted that due to the severe browning and necrosis of GT1 anther callus in the R73397 induction medium, different PGR formulations were required for the ‘induce’ stage to obtain a viable GT1 callus. However, during the subsequent somatic embryo induction stage (rediff), both the GT1 and R73397 calluses were cultured on the identical R73397 embryo induction medium. Despite being under identical induction conditions, GT1 still failed to undergo embryogenic transition, and the transcriptomic profile consistently lacked the activation of “anatomical structure development” pathways. Furthermore, no significant enrichment was observed in typical hormone-responsive pathways (e.g., responding to auxin), which suggests that differences in genotype have a greater impact on gene expression. As an exploratory analysis, we conducted a cross-stage differential gene analysis between GT1-induce and R73397-rediff, followed by a GO enrichment analysis of the downregulated genes (i.e., those highly expressed in GT1) (Figure 4C). Contrary to our expectations, the “embryo development” pathway was significantly enriched in this set of downregulated genes (Figure 4C). Based on this observation, we speculated that this might imply the possibility that embryogenic cells already exist within the GT1 anther callus, enabling the expression of certain genes required during the initial stages of somatic embryogenesis. Conversely, in R73397, the embryogenic cells might have already progressed past the initial stages to develop anatomical structures characteristic of the typical globular stage during somatic embryo induction; consequently, the expression of early embryogenesis genes was downregulated. It should be emphasized that this logical deduction currently serves purely as an exploratory hypothesis, intended to provide insights for future research, and requires further rigorous experimental validation. On another hand, the biological process “reproductive process” was more active in GT1 during the somatic embryo induction stage, which indicates a strong propensity for somatic embryogenesis.

Compared with previous similar studies [26,31], our transcriptome analysis does not reveal significant enrichment in auxin biosynthesis-related metabolic pathways or MAPK signaling pathways. The GO enrichment analysis indicates that the GT1 callus expresses several early embryogenesis genes, though embryo development is arrested prior to the formation of typical globular embryos. Based on these findings, we propose that the bottleneck in GT1 somatic embryogenesis lies not only in embryogenic initiation, but also in the morphogenetic transition phase. Even if totipotent cells exist, they fail to complete the differentiation and remodeling processes required to transition from a single cell into a complex multicellular structure. During the somatic embryo induction stage, the “reproductive process” in GT1 is active, and the “embryo development” pathway is in a relatively activated state. This means that for strategies aimed at optimizing the somatic embryogenesis rate of GT1 callus, focusing on the process by which embryonic cells develop into embryonic morphology is just as important as focusing on the generation of cellular embryogenic potential.

GT1 seeds develop normally, and somatic embryogenesis occurs sporadically in the anther callus, indicating that the essential genes are largely intact within the genome but fail to be properly activated. All 127 key-TFs identified in our screen exhibited direct or indirect binding associations with the “post-embryonic development” process. This indicates that silencing of such kinds of genes related to post-embryonic development in GT1 is one of the core reasons for the impaired somatic embryo development.

Cytosine DNA methylation is a crucial epigenetic modification leading to gene silencing; however, this study found no strong correlation between these key transcription factors and DNA methylation. The five key-TFs harboring differentially methylated regions (Figure 5D,E) were annotated as scarecrow-like protein 14, NAC domain-containing protein 6, zinc finger protein ZAT10, asymmetric leaves 2-like protein, and transcription factor bHLH130. Scarecrow-like protein 14 can be induced by 2,4-D and functions in the activation of stress-inducible promoters [32,33]; although NAC domain transcription factors are predominantly involved in callus induction and proliferation [34], there is no evidence that NAC6 directly regulates somatic embryogenesis. ZAT genes participate in abiotic stress responses, and while they play an important role in maintaining callus cell viability [35], they are not considered to control embryo development; although ectopic expression of certain LBD genes can induce plant regeneration [36], such reports do not involve AS2 or its homologs. bHLH transcription factors participate in biological processes, including embryogenesis and somatic embryogenesis [31], but they do not occupy a core position within the gene regulatory network governing somatic embryo development [37].

The TE regions in R73397 exhibited high CHH methylation alongside high chromatin accessibility, whereas GT1 displayed low TE methylation and low accessibility. Typically, high methylation is associated with low accessibility [38]. However, while maintaining high TE methylation to silence the transposable elements, R73397 concurrently preserved high chromatin accessibility at neighboring genes. This indicates that different rubber tree varieties employ distinct strategies to achieve TE silencing, a perspective that warrants close attention in future research on other traits of rubber trees.

Similar to the case of differential methylation, the differential expression of the 127 key-TFs showed only a weak correlation with chromatin accessibility. Two genes known to be strongly associated with somatic embryogenesis that harbor DARs are *LOC110633278* (*WOX8*) and *LOC110635766* (*NF-YA3*). *WOX8* is one of the master regulators of embryo development, expressed in early embryos shortly after fertilization [37]. This corresponds precisely to the phase where a single cell develops into an embryo with anatomical structures, aligning with the several biological processes found to be missing in GT1 according to our DEG enrichment results. NF-Y transcription factors typically function by forming a heterotrimeric complex comprising the NF-YA, NF-YB, and NF-YC subunits [37]. In the field of somatic embryogenesis, the most extensively studied NF-YB gene is *LEC1* [12]. However, as an integral component of the trimeric complex, NF-YA is equally indispensable. Despite this, the chromatin accessibility of these two genes was lower in R73397, which contrasts with their high expression levels. Given that their less accessible DARs are located immediately adjacent to the TATA box, we propose that these regions are occupied by transcribing RNA polymerase, thereby interfering with chromatin accessibility detection. Consequently, the observed decrease in accessibility is a direct result of active transcription [39].

RKD4 is a transcription factor capable of independently inducing somatic embryogenesis [37,40], holding an importance comparable to BBM/PLT in the AP2 family, WUS/WOX in the homeobox family, and LEC1 in the NF-Y family. We highlight these classes of genes within the regulatory network predicted based on the Tn5 transposase footprints. Reported cases of MYB44 regulating somatic embryogenesis are limited. In upland cotton (*Gossypium hirsutum* L.), although *GhMYB44* overexpression hinders callus growth, it promotes the differentiation of embryogenic callus [41]. Therefore, we include it in our annotation. However, the function of MYB44 is not conserved across different plant species; thus, we recommend it as a secondary target for subsequent research. Similarly, while genes such as *NF-YA1*, *NF-YA6*, *NF-YA9*, and *NF-YA10* share high homology with *NF-YA3*, there is a lack of evidence demonstrating their direct positive regulation of somatic embryogenesis. Therefore, they are not recommended as priority targets for investigation. Ultimately, we propose two *BBM* genes, two *WOX* genes, one *NF-YA3* gene, and one *RKD4* gene as target candidates for subsequent genetic engineering approaches aimed at enhancing the somatic embryogenesis rate in GT1. All of these genes are positioned at the top tier of the transcription factor regulatory network, with three of them annotated within the “anatomical structure development” process.

## 4. Materials and Methods

### 4.1. Plant Materials

Flowering shoots of the clonal rubber tree (*Hevea brasiliensis*) cultivar R73397 were collected from healthy, mature trees cultivated at the experimental field of the Rubber Research Institute, Chinese Academy of Tropical Agricultural Sciences, Danzhou, Hainan Province, China.

Flowering shoots of the clonal rubber tree (*Hevea brasiliensis*) cultivar GT1 were collected from healthy, mature trees cultivated near the Licun Reservoir (109.34 east longitude, 18.38 north latitude), Sanya, Hainan Province, China.

All the calluses used for sequencing in this study were from anthers that were collected on 11 March 2025. To ensure independent validation, we recollected rubber tree anthers on 7 March 2026 and repeated the entire tissue culture procedure de novo; the 2nd batch of resulting calluses was then subjected to qRT-PCR analysis.

### 4.2. Whole-Genome Resequencing (WGS)

No biological or technical replicates were performed for this assay. DNA extraction, database building, sequencing, data quality control, and data cleaning were completed by Berry Genomics (Beijing, China). DNBSEQ-T7-Sequencing platform (MGI TECH Co., Ltd., Shenzhen, China) was used. Clean sequencing data from multiple times of database building were merged to ensure final sequencing depth for each line was over 20×. Clean reads were aligned to reference rubber tree genome (GCF_030052815.1_ASM3005281v1) using BWA [42]. Processing files were parsed using SAMtools [43], and mutations, including single-nucleotide polymorphisms (SNPs) and small insertions and deletions (InDels), were identified using BCFtools [44]. Principal components of SNP/InDel were analyzed with Plink [45]. GT1 and other cultivars’ WGS reads were obtained from previous published data [1,46,47].

### 4.3. Anther-Induced Callus Induction Method and Culture Medium

The callus and somatic embryo induction of R73397 used a previously published protocol [27].

The callus and somatic embryo induction and subculture of GT1 were further improved based on a previously published protocol. The catalog numbers and manufacturers of the reagents used are listed in Table 1.

All anther and callus tissues were maintained in a dark incubator at a constant temperature of 26 °C.

### 4.4. RNA Sequencing

Three biological repetitions were performed for each line. RNA extraction, database building, sequencing, and data quality control were completed by Berry Genomics (Beijing, China). The DNBSEQ-T7-Sequencing platform (MGI TECH Co., Ltd., Shenzhen, China) was used, with sequencing data of over 6 GbP for each replicate. The clean reads were aligned to the reference genome using Hisat2 (version 2.2.1) with default parameters [48]. The gene expressions were quantified using StringTie (version 2.2.3) [49], with parameters ‘-B’ and ‘-e’ to get TPM and ht-seq (version 2.0.5) [50], and with default parameters to get the read counts. The gene expression difference analyses were performed using the R package DESeq2 (version 1.52.0) [51] with default parameters. The resulting *p*-values were adjusted for multiple testing using the Benjamini–Hochberg method to control the false discovery rate (FDR). Genes with values of *Padj* ≤ 0.01 and |log_2_FC| ≥ 1.0 were considered differentially expressed genes (DEGs). When calculating the Fold Change of GT1-induce vs. R73397-induce, the gene expression of GT1-induce was used as the denominator, and the gene expression of R73397-induce was used as the numerator. Differential genes with log_2_(FC) > 0 were upregulated in R73397-induce. When calculating the Fold Change of GT1-rediff vs. R73397-rediff, the gene expression of GT1-rediff was used as the denominator, and the gene expression of R73397-rediff was used as the numerator. Differential genes with log_2_(FC) > 0 were upregulated in R73397-rediff. When calculating the Fold Change of GT1-induce vs. R73397-rediff, the gene expression of GT1-induce was used as the denominator, and the gene expression of R73397-rediff was used as the numerator. Differential genes with log_2_(FC) > 0 were upregulated in R73397-rediff. Enrichment analyses were performed using TBtools (version 2.472) [52] with the gene-annotation of eggNOG (version 2.1.12) [53] using the parameter ‘--translate’. All genes that were detected with a non-zero expression at least once in the four kinds of callus tissues were used as the background for the enrichment analysis. The *p*-values were calculated using a hypothesis test based on the hypergeometric distribution, and were adjusted for multiple testing using the Benjamini–Hochberg method to control the false discovery rate.

### 4.5. Whole-Genome Bisulfite Sequencing (WGBS)

Three biological repetitions were performed for each line. The DNA bisulfite treatment, database building, sequencing, and data quality control were completed by Berry Genomics (Beijing, China) using the Illumina Novaseq6000-Sequencing platform, with a sequencing depth of over 20× for each line. The acquired sequence data were aligned to the reference genome with bwa-meth (version 0.2.9) (arXiv:1401.1129 [q-bio.GN]) with default parameters, and the methylation level was analyzed using Methydackel (version 0.6.1) (https://github.com/dpryan79/MethylDackel (accessed on 11 July 2024)) with parameters ‘—CHG’ and ‘--CHH’ to generate any contexts of methylation level. The methylation differences were analyzed with Metilene (version 0.6.9) (http://legacy.bioinf.uni-leipzig.de/Software/metilene/ (accessed on 11 July 2024)) with default parameters. DNA methylation in the three sequence contexts (CG, CHG, and CHH) was analyzed separately. Candidate DMRs were first delineated by a circular binary segmentation algorithm on the mean methylation difference signal, then evaluated using a two-dimensional Kolmogorov–Smirnov test (2D-KS test) and a Mann–Whitney U test. The resulting *p*-values were adjusted for multiple testing using the Benjamini–Hochberg method to control the false discovery rate (FDR). Regions with DNA methylated level of *Padj* less than 0.01 and a difference greater than 0.3 were considered differentially methylated regions (DMRs).

### 4.6. Assay for Transposase-Accessible Chromatin with High-Throughput Sequencing (ATAC)

Two biological repetitions were performed for each line. Nuclear extraction, database building, sequencing, and data quality control were completed by Wuhan Carbon Code Biotechnologies (Wuhan, China) using the Illumina Novaseq6000-Sequencing platform with a sequencing depth of over 20× for each line. The acquired sequence data were aligned to the reference genome with BWA-MEM (version 0.7.18) [42] with default parameters, and shifted with deeptools-alignmentSieve (version 3.5.5) [54] with default parameters. The accessibility difference was analyzed with PePr (version 1.1.24) [55], with the parameter ‘-n ATAC_diff’ to switch into ATAC mode. The read counts within sliding windows were modeled using a negative binomial distribution, with dispersion estimated locally from the surrounding genomic regions. Statistical significance was assessed by the negative binomial test, and the resulting *p*-values were adjusted for multiple testing using the Benjamini–Hochberg method to control the false discovery rate (FDR). Due to the fact that only two biological replicates were measured, a dual-criteria approach was used to define the differential regions: regions with an FDR below 0.01 and |log2FC| ≥ 1.0 were considered chromatin difference accessibility regions (DARs).

### 4.7. TF-DNA Binding Network Prediction

Transcription factor DNA-binding motifs were predicted with footprintDB (02/07/2026 Updated) [56,57]. The transcription factor footprints on the genome were analyzed with TOBIAS (version 0.17.0) and mapped to the predicted motifs following the pipeline and parameters recommended in its official guidelines [39].

### 4.8. qRT-PCR

The total RNA was extracted using a FlaPure Plant Total RNA Extraction Plus Kit (RE713, Genesand, Beijing, China) and reverse transcribed to cDNA using a UnionScript First-strand cDNA Synthesis Mix for qPCR with dsDNase (SR511, Genesand, Beijing, China). The reverse-transcribed cDNA at 1:10 served as the template. The GS AntiQ qPCR SYBR Green Fast Mix (SQ410, Genesand, Beijing, China) was used for qRT-PCR amplification. For both GT1 and R73397, three biological replicates were included. In addition, *HbACTIN* was used as the internal reference gene, like in the previous study [58].

The relative expression of each gene was calculated using the formula RE = 2−ΔΔCt, where CT is the cycle threshold, representing the number of cycles required for the fluorescent signal in the reaction tube to reach the threshold; ΔCt = the CT value of the target gene—the average CT value of the internal reference gene; ΔΔCt = ΔCT (sample 1) − ΔCT (sample 2); and the gene expression in GT1 was used as a control. All the primers are listed in Table 2.

## 5. Conclusions

The alternating application of GT1 callus induction medium and GT1 subculture medium enabled the repeated subculture and proliferation of GT1 anther callus.

The GO enrichment analysis of differentially expressed genes indicated the GT1 callus tissue had not completely lost the expression of genes that characterize the tendency to develop into embryos; however, these cells failed to express the genes required for the mid and late stages of embryo development, which should be considered as one of the core reasons why are they unable to develop into somatic embryos.

The differential expression of key transcription factors between the GT1 and R73397 calluses showed no strong correlation with either the genomic DNA methylation levels or chromatin accessibility.

The transposable elements (TEs) in R73397 exhibited higher average CHH methylation levels alongside higher average chromatin accessibility; this indicates that there may be unknown differences in the mechanisms of silencing transposons between the two lines.

We predicted the TF-DNA binding network of key transcription factors on the key pathways.

## Figures and Tables

**Figure 1:**
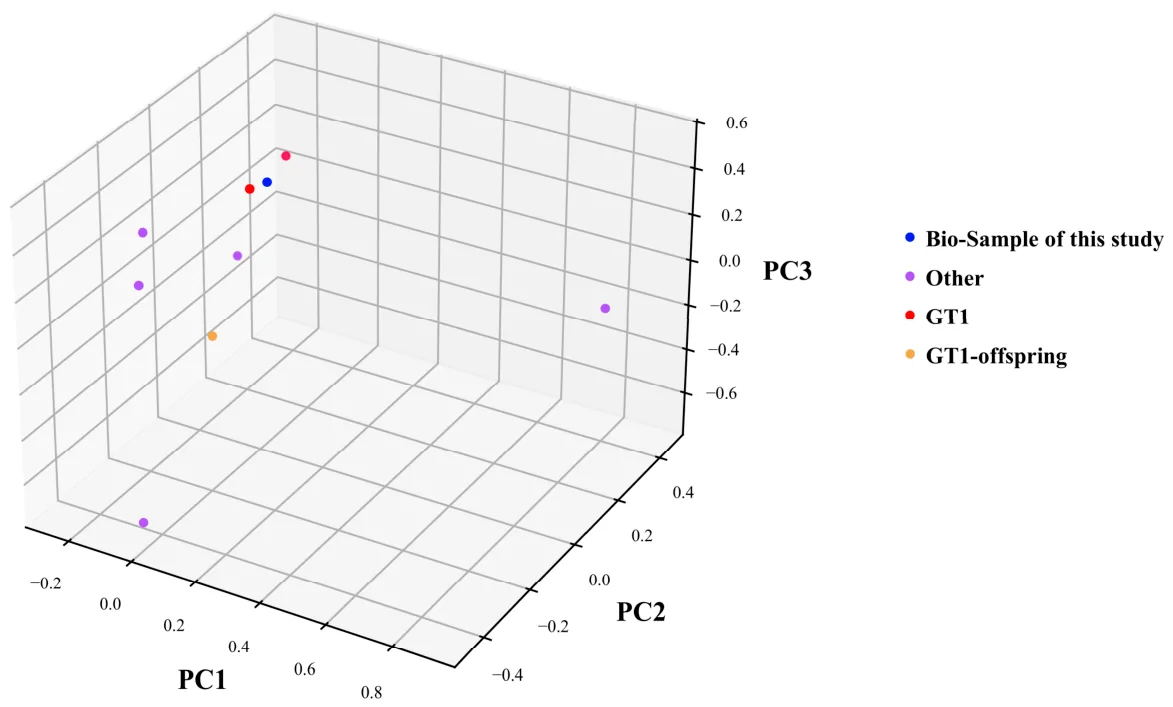
PCA results for SNP/InDel. Purple points represent PCA of CATAS8-333, PR107, RRIM600, RY879 and ReKen628.

**Figure 2:**
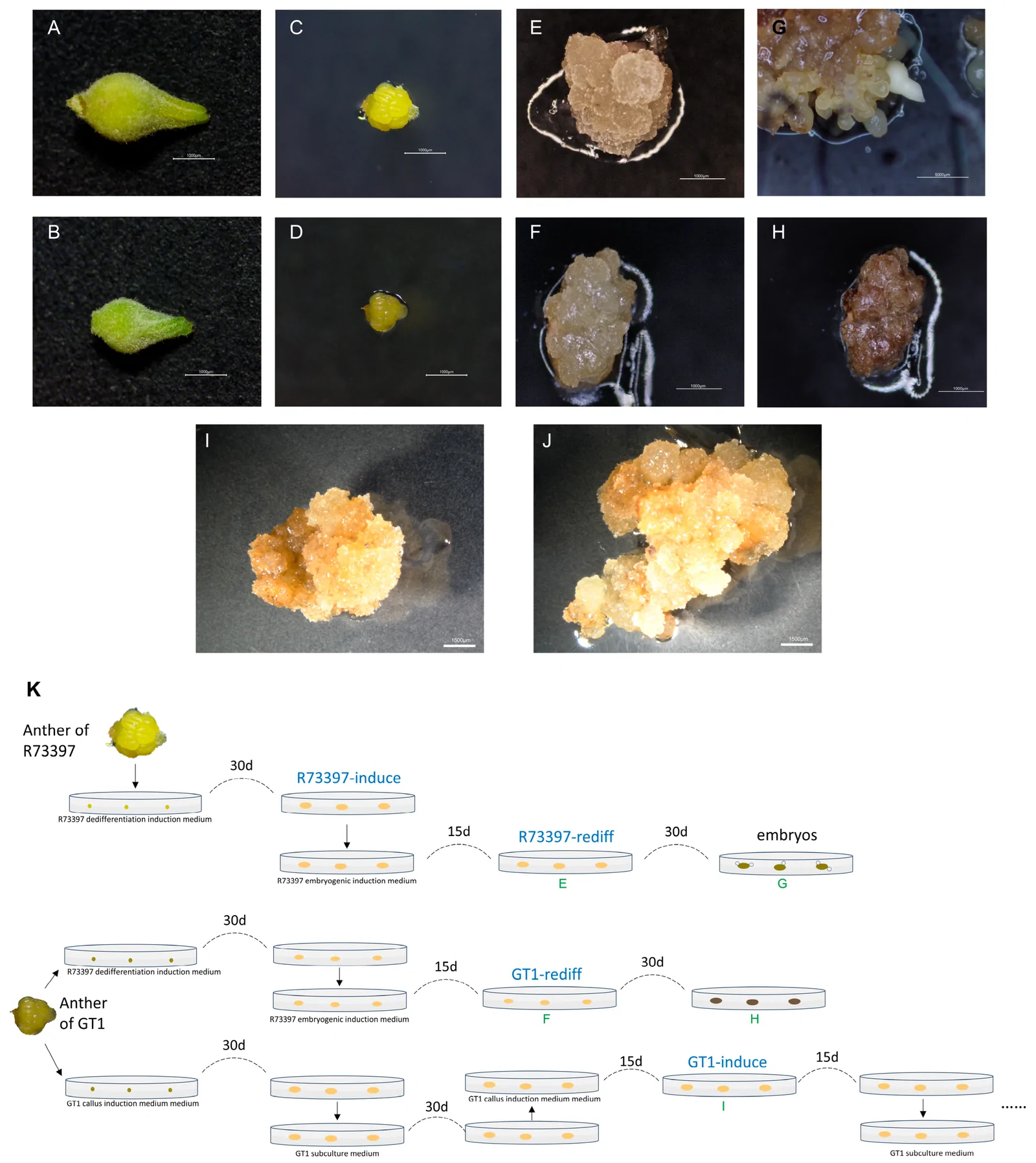
Comparison of anther and callus tissue between line R73397 and line GT1. (**A**) Male flower of R73397. Bar, 1 mm. (**B**) Male flower of GT1. Bar, 1 mm. (**C**) Anther of R73397. Bar, 1 mm. (**D**) Anther of GT1. Bar, 1 mm. (**E**) R73397 anther callus tissue obtained after 30 days of culturing on dedifferentiation induction medium and 15 d on embryogenic induction medium. Bar, 1 mm. (**F**) GT1 anther callus tissue obtained after 30 days of culturing on dedifferentiation induction medium and 15 d on embryogenic induction medium. Bar, 1 mm. (**G**) R73397 callus transferred to embryogenic induction medium for 45 days. Bar, 5 mm. (**H**) GT1 callus transferred to embryogenic induction medium for 45 days. Bar, 1 mm. (**I**) Callus after one round of alternating cultures. Anther material was first cultured with GT1 callus induction medium for 30 days to obtain callus, then cultured with GT1 subculture medium for 30 days, followed by GT1 callus induction medium for 15 days. Bar, 1.5 mm. (**J**) Callus after two rounds of alternating cultures. The callus from I continued to be cultured with GT1 callus induction medium for another 15 days, then with GT1 subculture medium for 30 days, and finally with GT1 callus induction medium for 20 days. Each time the medium was changed, these calluses were cut into small pieces ranging from 2 to 5 mm. Bar, 1.5 mm. (**K**) Schematic diagram of the tissue culture process showing the culture stages of E~I. Arrow, culture the material on the medium or switch to another type of medium; dashed line, time of culturing; blue text annotation, materials for subsequent NGS sequencing; green text annotation, stage of photographs above. GT1 callus induction medium, MS medium supplemented with vitamins 4.4 g/L; Phytagel 2.3 g/L; Sucrose 70 g/L; 6-BA 1 mg/L; 2,4-D 0.5 g/L; GA3 0.5 g/L; and pH = 5.8. GT1 subculture medium, MS medium supplemented with vitamins 4.4 g/L, Phytagel 2.3 g/L, Sucrose 70 g/L, KT 2 mg/L, NAA 2 mg/L, TDZ 0.2 mg/L, and pH = 5.8.

**Figure 3:**
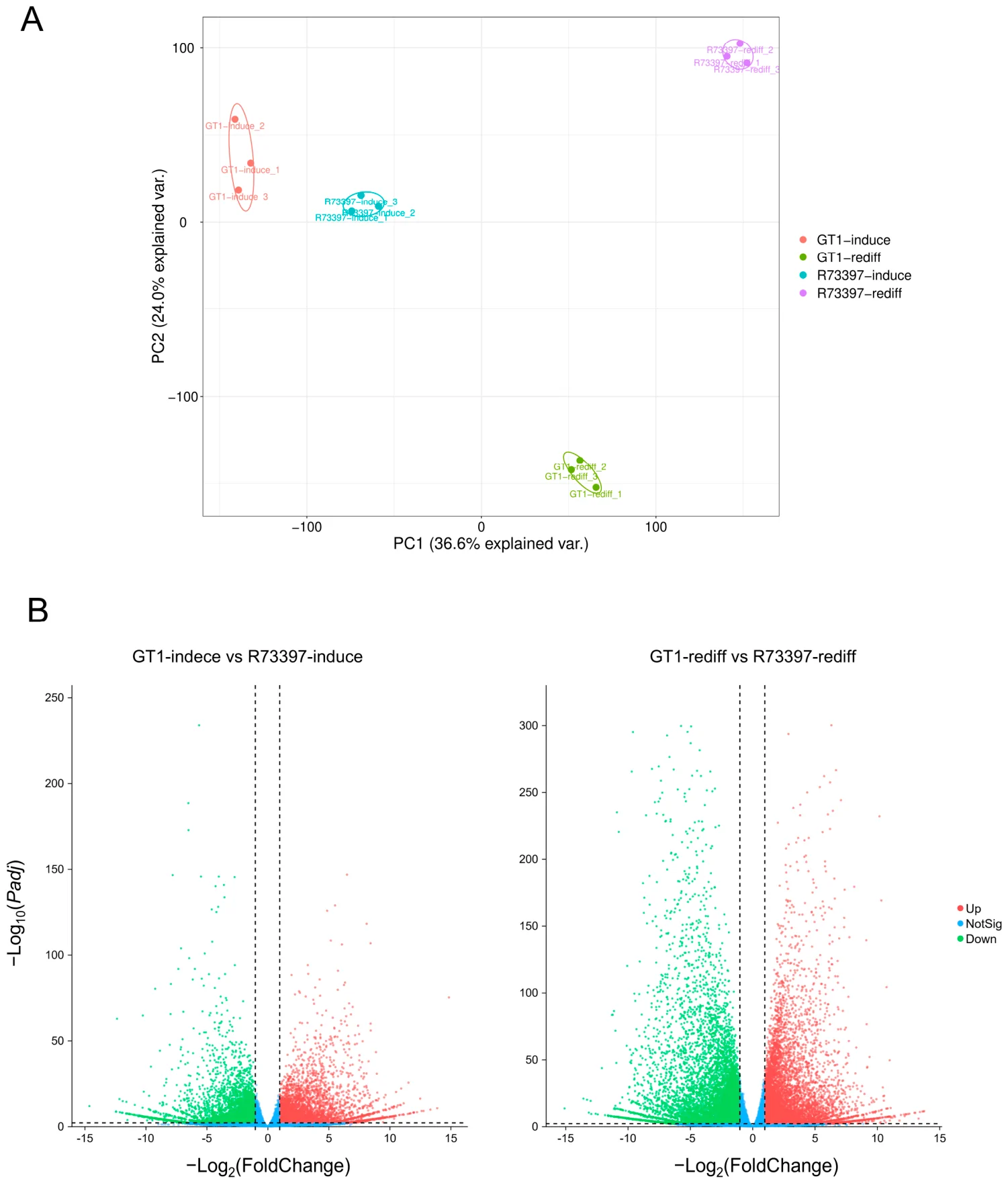
Principal component analysis and the volcano plot of DEGs. (**A**) PCA results of 4 biosamples’ gene expression patterns. (**B**) Volcano plot of DEGs of 2 stages. Differential expression analysis is performed using DESeq2. The resulting *p*-values are adjusted for multiple testing using the Benjamini–Hochberg method to control the false discovery rate (FDR). When calculating the Fold Change of GT1-induce vs. R73397-induce, the gene expression of GT1-induce is used as the denominator, and the gene expression of R73397-induce is used as the numerator. When calculating the Fold Change of GT1-rediff vs. R73397-rediff, the gene expression of GT1-rediff is used as the denominator, and the gene expression of R73397-rediff is used as the numerator. Genes with values of Padj ≤ 0.01 and |log2FC| ≥ 1.0 are considered differentially expressed genes (DEGs).

**Figure 4:**
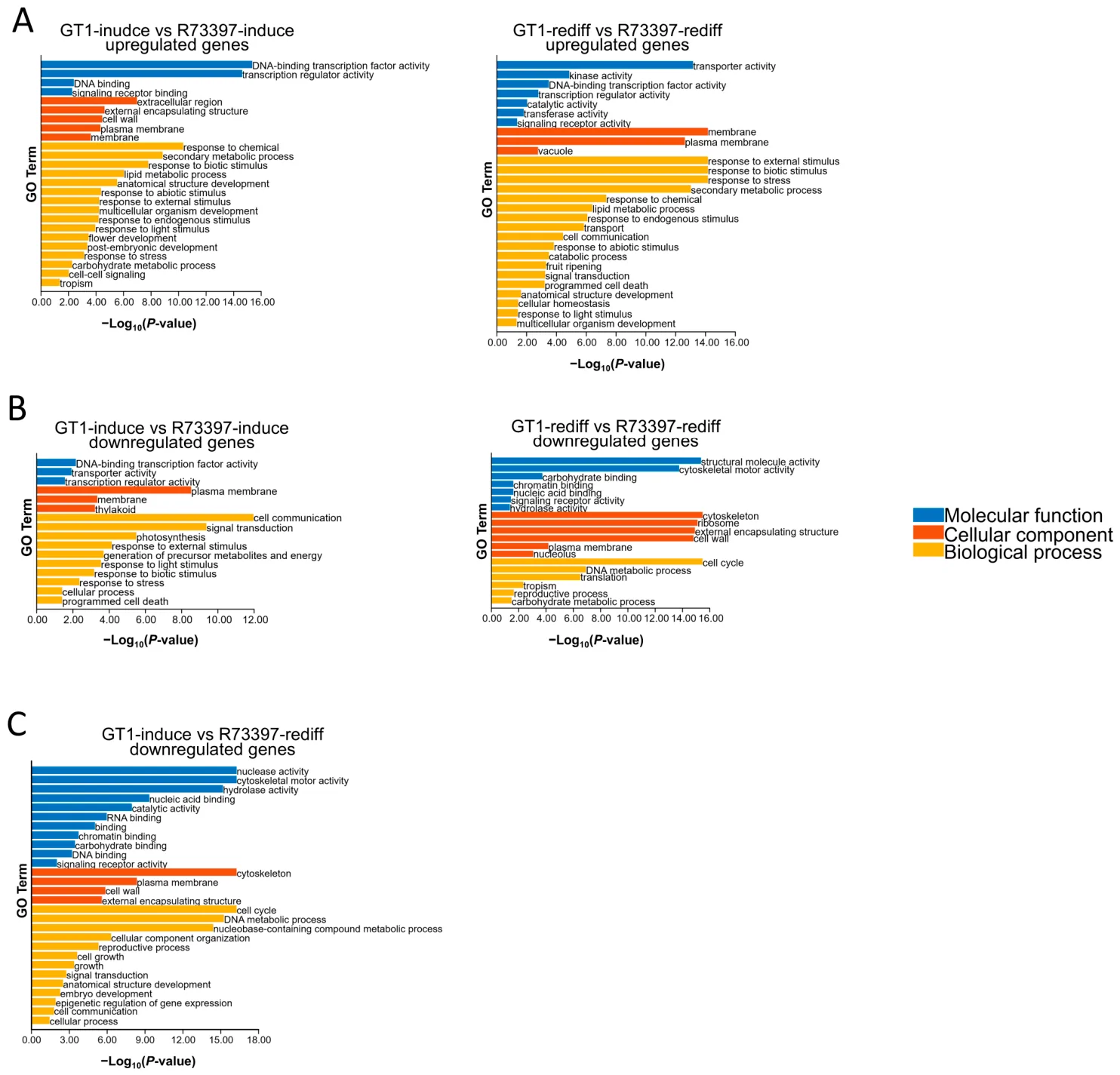
GO enrichment analysis results of differentially expressed genes in callus tissues of two lines at different stages. (**A**) GO enrichment of genes upregulated in R73397: GT1-induce vs. R73397-induce upregulated genes—DEGs with higher expression levels in R73397-induce than GT1-induce; GT1-rediff vs. R73397-rediff upregulated genes—DEGs with higher expression levels in R73397-rediff than GT1-rediff. (**B**) GO enrichment of genes downregulated in R73397: GT1-induce vs. R73397-induce downregulated genes—DEGs with lower expression levels in R73397-induce than GT1-induce; GT1-rediff vs. R73397-rediff downregulated genes—DEGs with lower expression levels in R73397-rediff than GT1-rediff. (**C**) GO enrichment of GT1-induce vs. R73397-rediff downregulated genes (DEGs with lower expression levels in R73397-rediff than GT1-induce).

**Figure 5:**
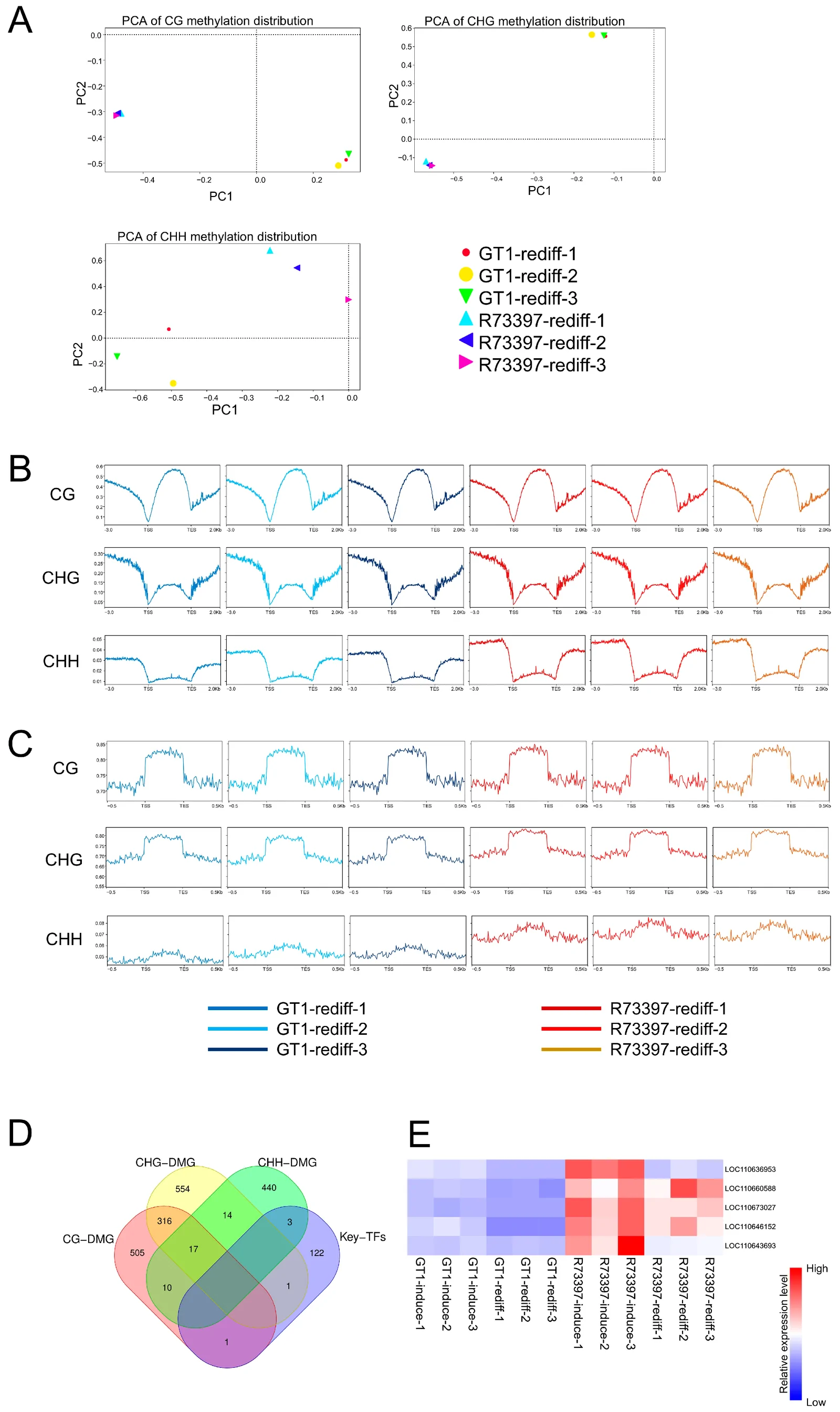
WGBS results. (**A**) PCA of the genomic methylation profiles. (**B**) Profile of DNA methylation distribution within gene regions. (**C**) Profile of DNA methylation distribution within TE regions. (**D**) Venn plot of differentially methylated genes (DMGs) and key-TFs. (**E**) Heatmap of relative expression levels of key-TFs that carry DMRs.

**Figure 6:**
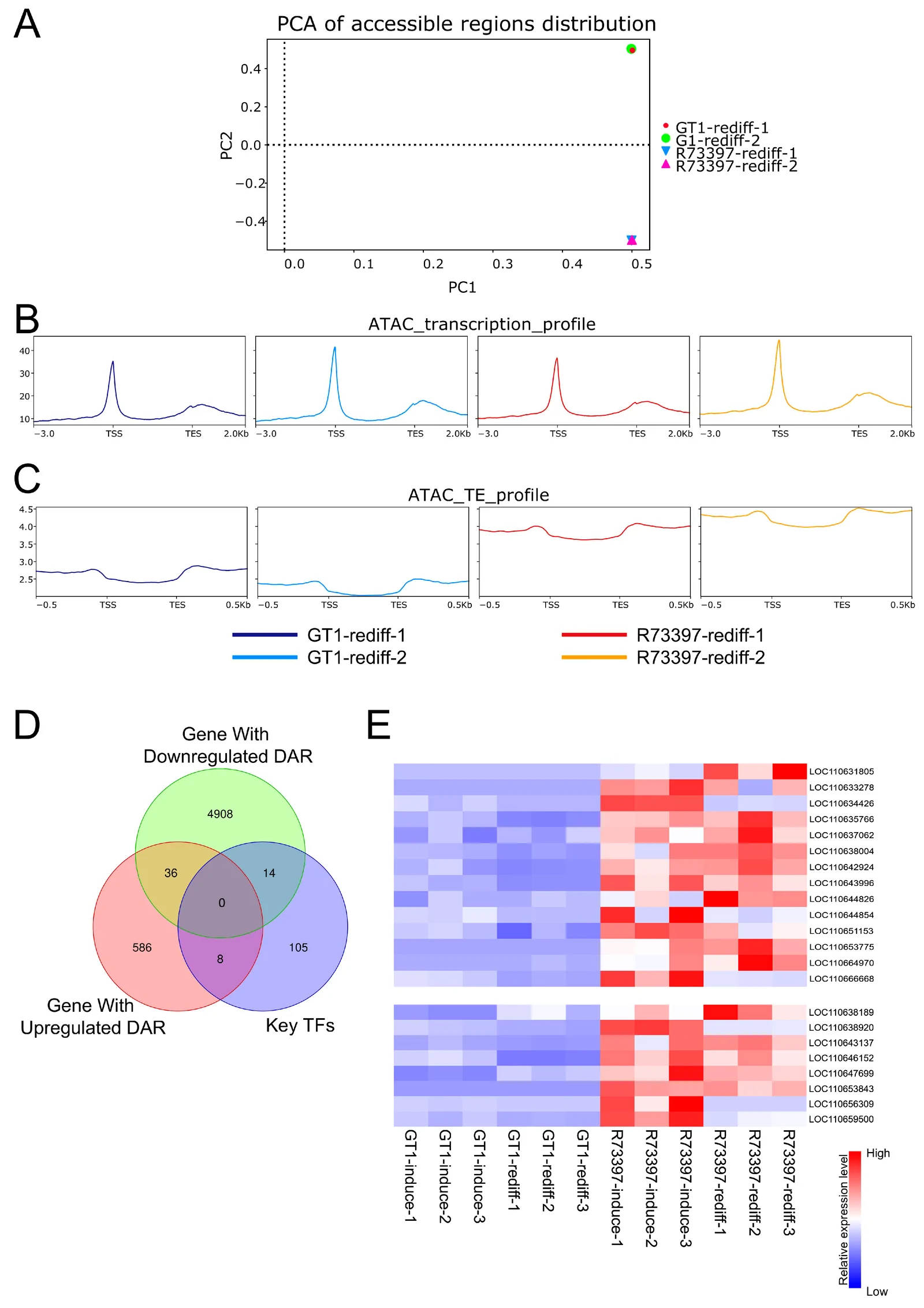
ATAC results. (**A**) PCA of the chromatin accessibility profiles. (**B**) Profile of chromatin accessibility within gene regions. (**C**) Profile of chromatin accessibility within TE regions. (**D**) Venn plot of genes carrying DAR and key-TFs. (**E**) Heatmap of relative expression levels of key-TFs that carry DARs.

**Figure 7:**
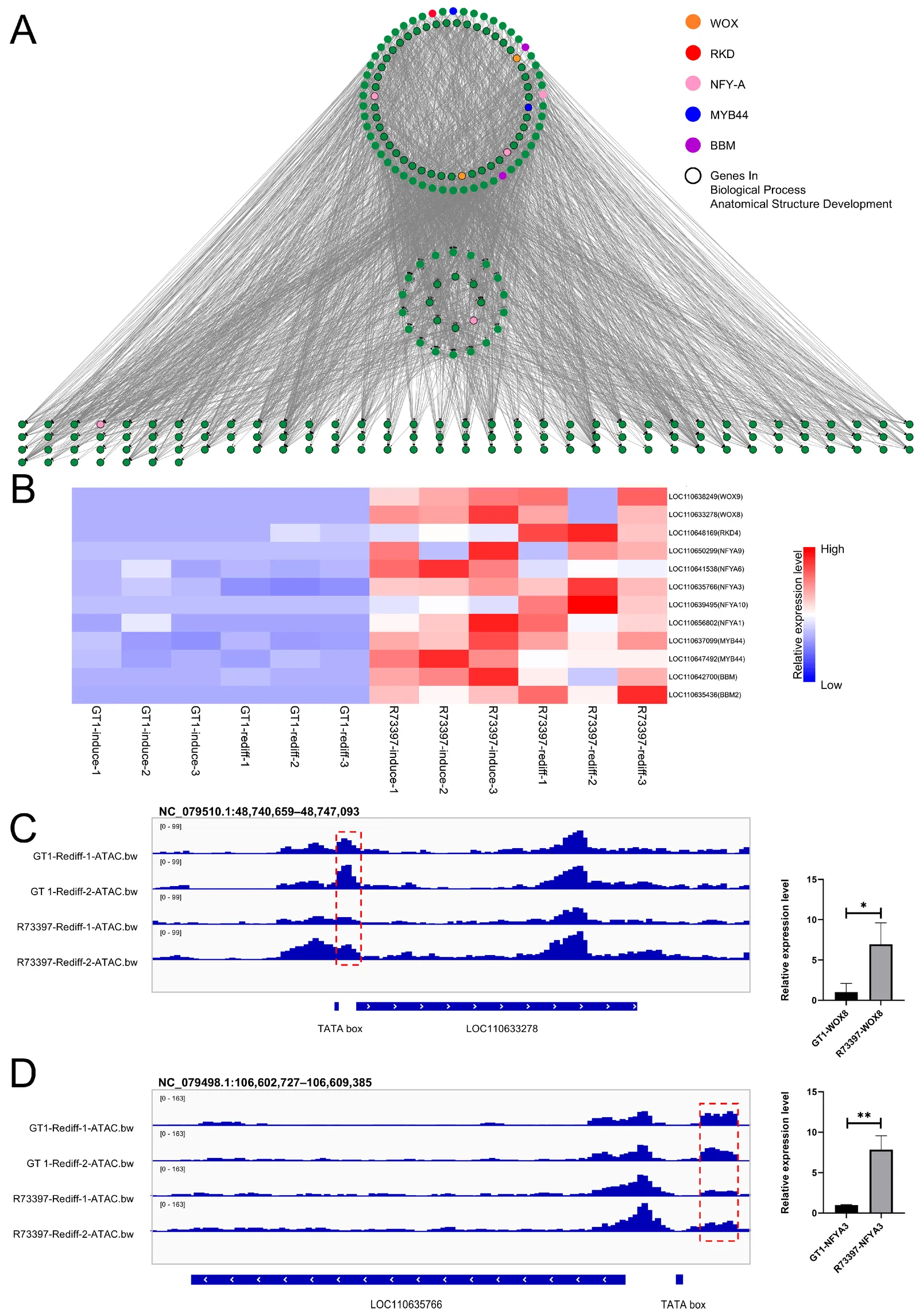
TF-DNA binding networks of key-TFs and DEGs involved in the “anatomical structure development” process. (**A**) Predicted binding relationships between genes: The arrows represent the protein of the source gene binding to the transcriptional regulatory regions of its target gene. The genes previously reported to explicitly promote secondary somatic embryogenesis are highlighted with non-green colors. The circle represents 127 key transcription factors (default color is green). (**B**) Heatmap of transcript level of highlighted genes. (**C**) Chromatin accessibility distribution and qRT-PCR results of LOC110633278 gene expression level. (**D**) Chromatin accessibility distribution and qRT-PCR results of LOC110635766 gene expression level. In the qRT-PCR for both the GT1-rediff callus and R73397-rediff callus, three biological replicates were included. The red dashed box represents chromatin difference accessibility regions (DAR). Error bars represent the SDs of three biological replicates. Asterisks indicate significant differences according to Student’s *t*-test (*, *p* ≤ 0.05; **, *p* ≤ 0.01).

**Table 1:** Culture medium for GT1.

Reagents	MS Base Salts	Phytagel	Sucrose	6-BA	KT	2,4-D	NAA	GA_3_	TDZ
Manufacturer	Coolaber, Beijing, China	Coolaber, Beijing, China	XiLONG SCIENTIFIC, Shenzhen, China	Coolaber, Beijing, China	Coolaber, Beijing, China	Coolaber, Beijing, China	Coolaber, Beijing, China	Coolaber, Beijing, China	Coolaber, Beijing, China
Catalog number	PM1011	CP8581Z	WXZY-ZT25KG	PH1041	PH111	PH1051	PH1011	PH1061	PH1071

**Table 2:** Primer sequences used for qRT-PCR.

Primer	Sequence
qLOC110648169-F	5′-attttgggtccgagggcttt-3′
qLOC110648169-R	5′-ttagtggctttgcgtcgact-3′
qLOC110638249-F	5′-gggccttgcactaaccttct-3′
qLOC110638249-R	5′-gccagactgatgctgtgagt-3′
qLOC110635436-F	5′-tcacaatcaggctcgccttt-3′
qLOC110635436-R	5′-ctcaatagcagcggtttggc-3′
qLOC110635766-F	5′-gaaaggctgagggaggtcac-3′
qLOC110635766-R	5′-gcctgttgtccatatgcagc-3′
qLOC110642700-F	5′-cctctcgcttaggcttccac-3′
qLOC110642700-R	5′-taccaaaagaggcagcgagg-3′
qLOC110633278-F	5′-tgtccaattcgccaactggt-3′
qLOC110633278-R	5′-tgacctagttcctcctccgg-3′
qHbACT-F	5′-caagggtgaatacgatgagtctg-3′
qHbACT-R	5′-gcctctcactagcagccataac-3′

## Data Availability

All NGS data in this manuscript is being uploaded to the China National Center For Bioinformation (https://www.cncb.ac.cn/), BioProject PRJCA070974.

## Supplementary Materials

The following supporting information can be downloaded at: https://www.mdpi.com/article/10.3390/plants15192924/s1, Supplementary Table S1: WGS-PCA.xlsx; Supplementary Table S2: KEGG enrichment result of GT1-induce vs R73397-induce; Supplementary Table S3: KEGG enrichment result of GT1-rediff vs R73397-rediff; Supplementary File S1: Fresh weight of GT1 callus and the proportion of actively proliferating callus among total GT1 callus; Supplementary File S2: GT1-rediff VS R73397-rediff KEGGenrichment.pdf; Supplementary File S3: GT1-rediff VS R73397-rediff KEGGenrichment; Supplementary Figure S1: Heatmap of key TFs’ relative expression levels; Supplementary Figure S2: Heatmap of key TFs TPM; Supplementary Figure S3: qPCR result of WOX9 NFYA BBM and BBM2.

## Abbreviations

The following abbreviations are used in this manuscript:

SE	Somatic Embryogenesis
WGS	Whole-Genome Sequencing
PCA	Principal Component Analysis
SNP/InDel	Single-Nucleotide Polymorphism/Insertion and Deletion
DEG	Differentially Expressed Genes
WGBS	Whole-Genome Bisulfite Sequencing
DMR	Differentially Methylated Region
DMG	Differentially Methylated Gene
ATAC	Assay for Transposase-Accessible Chromatin with High-Throughput Sequencing
DAR	Chromatin Difference Accessibility Region
TF	Transcription Factor
qRT-PCR	Real-Time Quantitative Polymerase Chain Reaction
6-BA	6-Benzylaminopurine
KT	Kinetin
2,4-D	2,4-Dichlorophenoxyacetic Acid
NAA	α-Naphthaleneacetic Acid
GA_3_	Gibberellin A3
TDZ	Thidiazuron
PGRs	Plant Growth Regulators
