# Supplementary material for: Optimized Callus Subculture and Multi-Omics Analysis Reveal Somatic Embryogenesis Bottlenecks in the Cold-Resistant Rubber Tree Clone GT1
Source: Plants (Basel). 2026 Sep 24;15(19):2924. doi: 10.3390/plants15192924 (PMC13666911; doi:10.3390/plants15192924)

In the GT1 tissue culture protocol used in this study, GT1 callus tissue were cut into small fragments (2–4 mm) during each subculture. To evaluate the biomass accumulation rate on GT1 subculture medium, we recorded the fresh weights of several initial callus pieces (ranging from 7.4 to 23.1 mg). Following 21 days of cultivation on GT1 subculture medium, the fresh weights of the resulting callus clumps were also measured (ranging from 66.9 to 170.4 mg). These data indicate that under our culture conditions, GT1 callus tissue can increase its biomass by approximately 6.5-fold within 21 days, even after more than four rounds of subculture. This proliferation rate is of the same order of magnitude as that observed in R73397 anther-derived callus tissue.

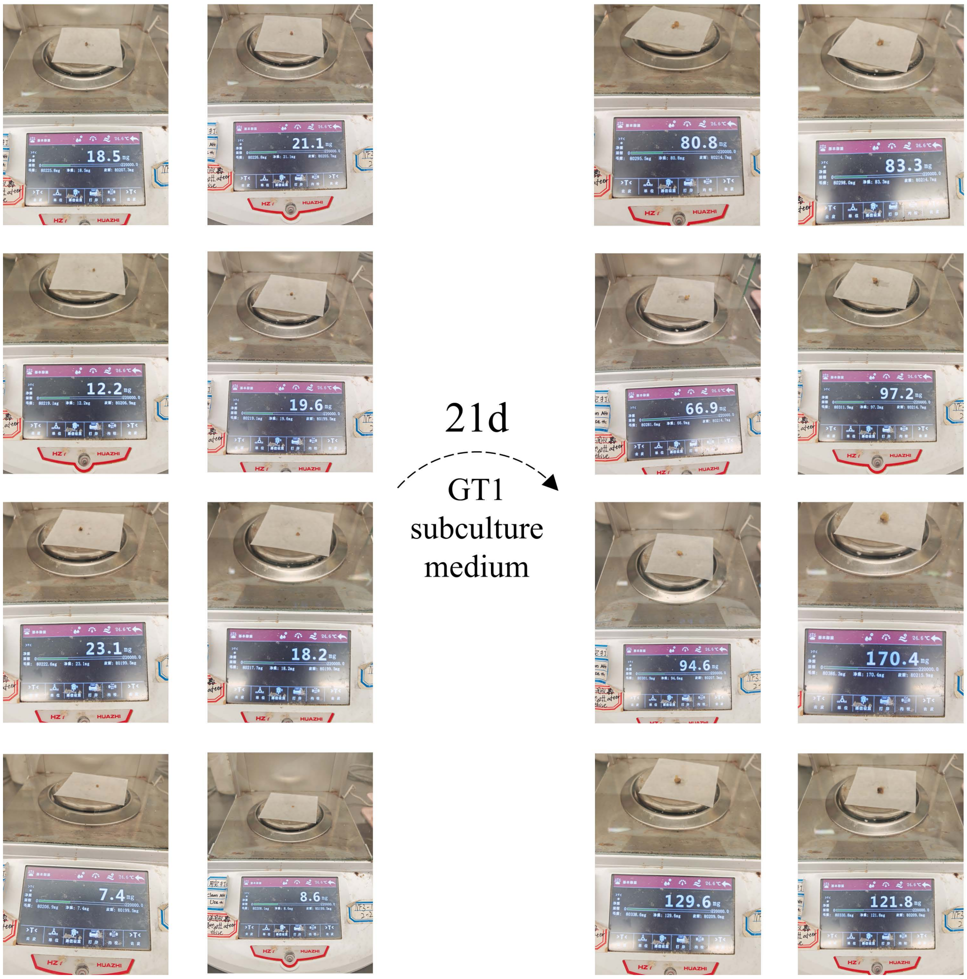

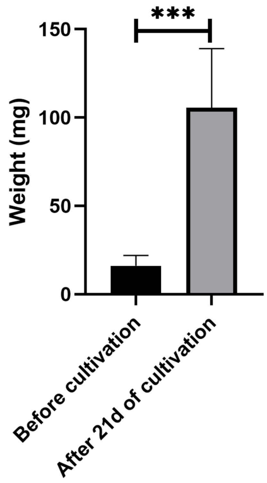

Asterisks indicate significant differences according to Student's t-test (∗ ∗ ∗, $P \leq 0.001$)

We have also evaluated the proportion of light-yellow, actively proliferating GT1 callus. 5 dishes of GT1 callus on GT1 subculture medium were selected randomly. The overall regeneration proportion of GT1 callus reached 80.95%, even after more than four rounds of subculture.

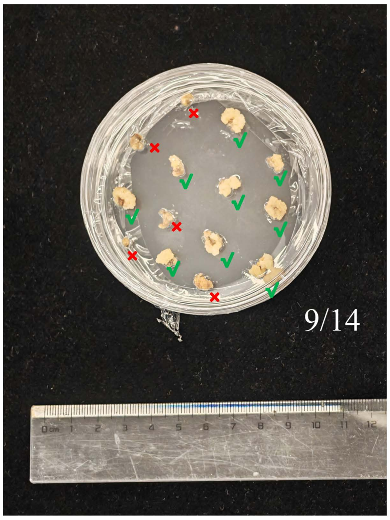

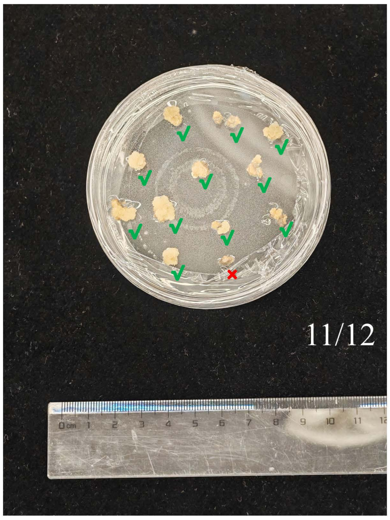

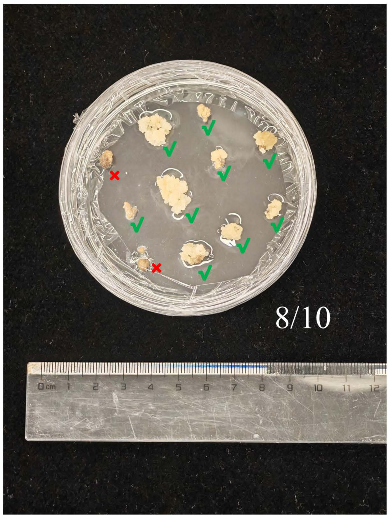

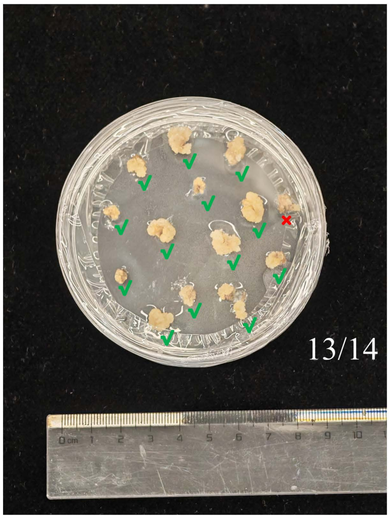

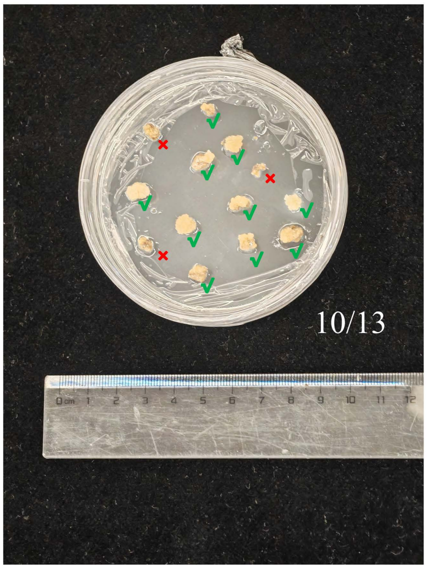

Supplement: Supplementary file 1 [file plants-15-02924-s001.zip › Supplementary_File_S1_Fresh_weight_of_GT1_callus_and_the_proportion_of_actively_proliferating_callus_among_total_GT1_callus.pdf]
