# Supplementary material for: Optimized Callus Subculture and Multi-Omics Analysis Reveal Somatic Embryogenesis Bottlenecks in the Cold-Resistant Rubber Tree Clone GT1
Source: Plants (Basel). 2026 Sep 24;15(19):2924. doi: 10.3390/plants15192924 (PMC13666911; doi:10.3390/plants15192924)
Supplement: Supplementary file 1 [file plants-15-02924-s001.zip › Supplementary_File_S2_GT1-induce_VS_R73397-Induce_KEGGenrichment.pdf]

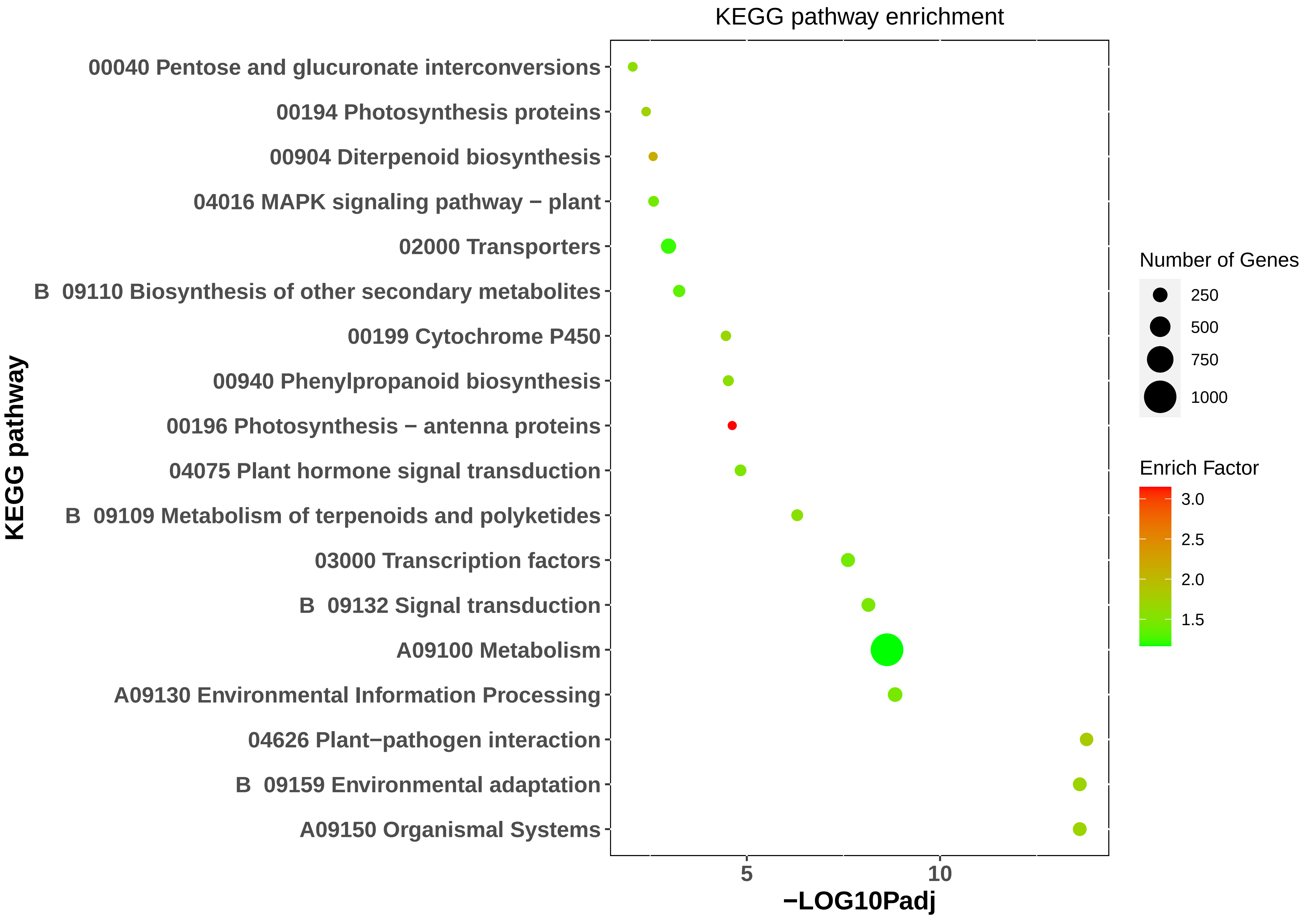
KEGG pathway enrichment
KEGG pathway
00040 Pentose and glucuronate interconversions
00194 Photosynthesis proteins
00904 Diterpenoid biosynthesis
04016 MAPK signaling pathway − plant
02000 Transporters
B 09110 Biosynthesis of other secondary metabolites
00199 Cytochrome P450
00940 Phenylpropanoid biosynthesis
00196 Photosynthesis − antenna proteins
04075 Plant hormone signal transduction
B 09109 Metabolism of terpenoids and polyketides
03000 Transcription factors
B 09132 Signal transduction
A09100 Metabolism
A09130 Environmental Information Processing
04626 Plant−pathogen interaction
B 09159 Environmental adaptation
A09150 Organismal Systems
5
10
−LOG10Padj
Number of Genes
250
500
750
1000
Enrich Factor
3.0
2.5
2.0
1.5
