# Supplementary material for: Optimized Callus Subculture and Multi-Omics Analysis Reveal Somatic Embryogenesis Bottlenecks in the Cold-Resistant Rubber Tree Clone GT1
Source: Plants (Basel). 2026 Sep 24;15(19):2924. doi: 10.3390/plants15192924 (PMC13666911; doi:10.3390/plants15192924)
Supplement: Supplementary file 1 [file plants-15-02924-s001.zip › Supplementary_File_S3_GT1-rediff_VS_R73397-rediff_KEGGenrichment.pdf]

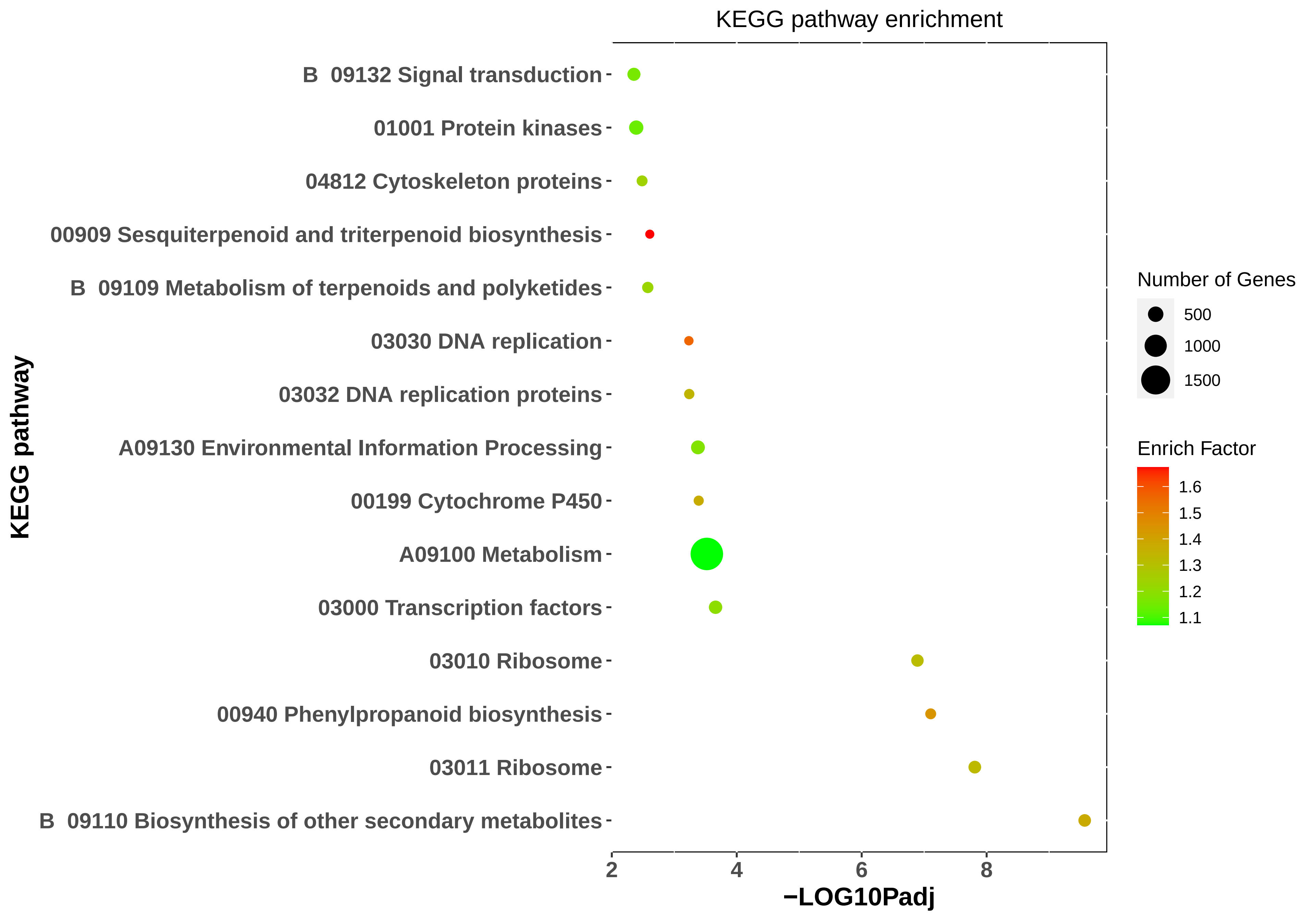

KEGG pathway enrichment
KEGG pathway
B 09132 Signal transduction
01001 Protein kinases
04812 Cytoskeleton proteins
00909 Sesquiterpenoid and triterpenoid biosynthesis
B 09109 Metabolism of terpenoids and polyketides
03030 DNA replication
03032 DNA replication proteins
A09130 Environmental Information Processing
00199 Cytochrome P450
A09100 Metabolism
03000 Transcription factors
03010 Ribosome
00940 Phenylpropanoid biosynthesis
03011 Ribosome
B 09110 Biosynthesis of other secondary metabolites
2
4
6
8
−LOG10Padj
Number of Genes
500
1000
1500
Enrich Factor
1.6
1.5
1.4
1.3
1.2
1.1
